# Bis­[*N*,*N*-bis­(di­phenyl­phosphan­yl)cyclo­hexa­namine-κ^2^*P*,*P*′]di­chlorido­cobalt(III) tris­(μ-di­phenyl­phos­phinato-κ^2^*O*:*O*′)bis­[chloridocobaltate(II)]–di­chloro­methane–diethyl ether (1/1.189/0.811)

**DOI:** 10.1107/S2414314625006327

**Published:** 2025-07-29

**Authors:** Sizwe J. Zamisa, Adesola A. Adeleke, Dunesha Naicker, Holger B. Friedrich, Bernard Omondi

**Affiliations:** a School of Chemistry and Physics, University of KwaZulu-Natal, Private Bag X54001, Durban, 4000, Republic of South Africa; Purdue University, USA

**Keywords:** crystal structure, Co(II)/Co(III) compound, amino­diphosphine, di­phenyl­phospho­nate

## Abstract

The DCM-Et_2_O solvated title compound, [CoCl_2_(C_30_H_31_NP_2_)_2_][Co_2_(C_12_H_10_O_2_P)_3_Cl_2_]·1.189CH_2_Cl_2_·0.811C_4_H_10_O or [CoCl_2_{Ph_2_PN(C_6_H_11_)PPh_2_}_2_][Co_2_{Ph_2_PO_2_}_3_Cl_2_]·1.189DCM·0.811Et_2_O, is a combination of a cationic Co^III^-amino­diphosphine and a dimetallic Co^II^-di­phenyl­phospho­nate anionic species. The asymmetric unit features one half of the cationic and anionic species with solvent mol­ecules in the outer coordination sphere. The metal centre of the cation is located on an inversion center, while the anion is disordered around an inversion center. The cyclo­hexa­nyl moiety of the amino­diphosphine ligand exhibits two-component rotational disorder along the N—C bond, the moiety’s major component occupancy refined to 71.1 (7)%. The di­chloro­methane and diethyl ether solvate mol­ecules are disordered with each other and were modelled with 59.4 (3)% and 40.6 (3)% site occupancies in the asymmetric unit, respectively. Inter­molecular C—H⋯π hydrogen-bonding patterns form infinite supra­molecular sheets extending parallel to (110). C—H⋯Cl and C—H⋯O hydrogen bonds were also found in the crystal packing of the title compound.

## Abstract 2

The solvated title compound has a cationic Co^III^-amino­diphosphine and a dimeric Co^II^-di­phenyl­phospho­nate anionic species.

## Graphical abstract

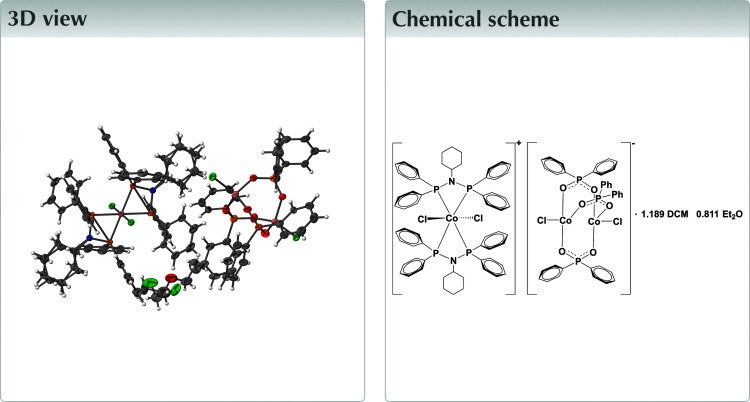

## Structure description

Nitro­gen-containing polyphosphines are known for their diverse structural motifs, versatile donating ability, and wide applications, especially in homogeneous catalysis (Parisel *et al.*, 2004 ▸; Wang *et al.*, 2013 ▸) and supra­molecular chemistry (Wang *et al.*, 2013 ▸). A commonly used ligand in this group is the flexible ‘PNP’ amino­diphosphine ligand, which has a nitro­gen atom that does not coordinate to the metal centre. Over the years, various derivatives of their transition-metal complexes have been synthesized (Benito-Garagorri & Kirchner, 2008 ▸; Merz *et al.*, 2020 ▸; Xiao *et al.*, 2020 ▸) where the substituents on the phospho­rus and nitro­gen atoms were derivatized, thus encouraging fine-tuning of their steric and electronic properties. Such modifications influence their catalytic activity, including the electrolytic activities (Xiao *et al.*, 2020 ▸) and promoting proton-coupled electron transfer in the catalytic reduction of protons (DuBois, 2014 ▸). Consequently, the ‘PNP’ ligand framework continues to gain attention when designing efficient catalysts with broad applications. In an effort to grow a crystal of a cobalt amino­diphosphine complex having a cyclo­hexyl ring on its nitro­gen atom as related to the continuation of our research on cobalt amino­diphosphine complexes as catalysts in the oxidation of *n*-octane (Naicker *et al.*, 2015 ▸), we determined the crystal structure of the title compound. It is suspected that the partial oxidation of the diphosphine ligand could have occurred during our crystal growth attempt, thus leading to the formation of the title compound.

**Figure 1:**
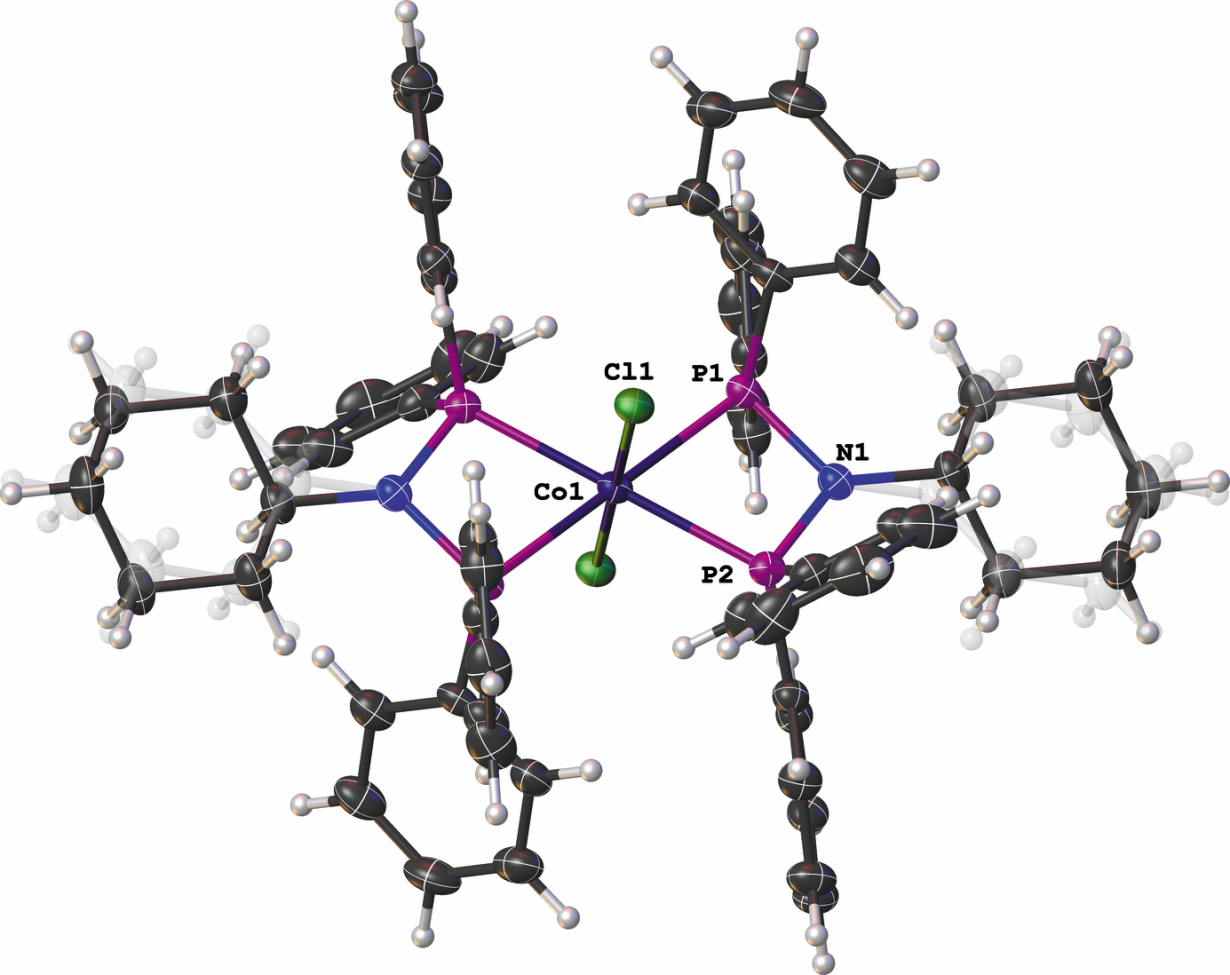
Molecular structure of the cationic species of the title compound with displacement ellipsoids drawn at the 50% probability level. The disordered cyclohexanyl moieties are drawn in a faded grey colour and the disordered solvent molecules have been omitted for clarity.

**Figure 2:**
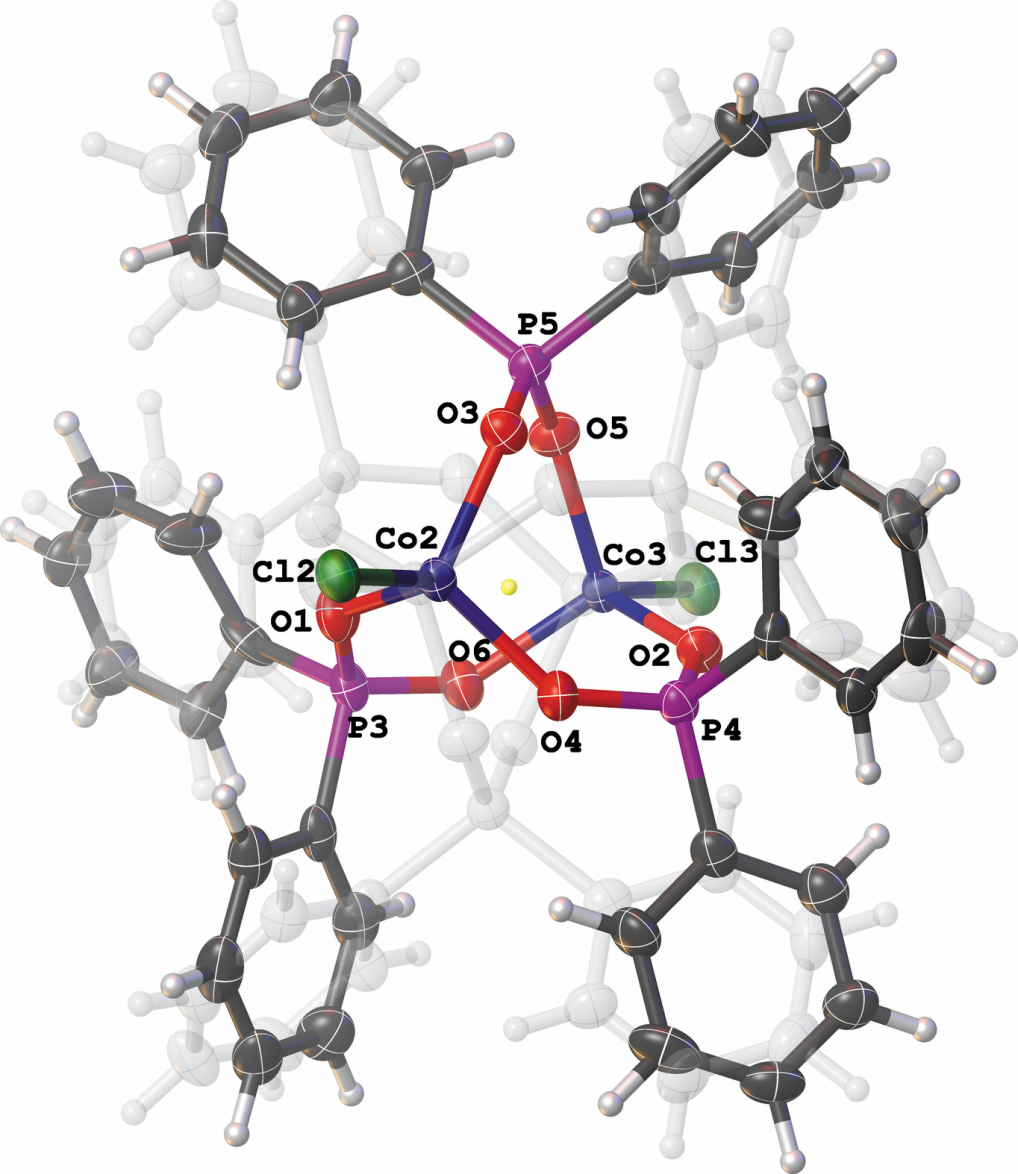
Molecular structure of the anionic species of the title compound with displacement ellipsoids drawn at the 50% probability level. The disordered second anionic moiety is drawn in a faded grey colour and the disordered solvent molecules have been omitted for clarity.

In the asymmetric unit of the title compound, there is half of a cationic cobalt(III) amino­diphosphine complex and one half of the dimetallic cobalt(II)-diphosphinite anionic complex, with di­chloro­methane and diethyl ether solvent mol­ecules. Moreover, the metal centre of the cation is located on an inversion center (symmetry operation: 1 − *x*, −*y*, −*z*), while the anion is disordered around an inversion center (symmetry operation: 1 − *x*, −*y*, 1 − *z*). In the structure of the cationic cobalt(III) amino­diphosphine species, the Co1 atom adopts a distorted octa­hedral geometry in which the Cl atoms occupy the axial positions of the octa­hedral vertices (Fig. 1 ▸). Two amino­diphosphine ligands, through the four P atoms, occupy the equatorial positions with acute P1—Co1—P2 bite angles [70.50 (2)°], which engender a short P1⋯P2 contact [2.6243 (8) Å] compared to the uncoordinated *N*-cyclo­hexyl-*N*-(di­phenyl­phosphan­yl)-1,1-di­phenyl­phosphanamine [P⋯P = 2.9750 (6) Å; Naicker *et al.*, 2016 ▸]. The coordination mode exhibited by the amino­diphosphine ligand is bidentate and forms an almost planar four-membered metallacycle defined by Co1—P1—N1—P2. In the anionic cobalt(II) diphosphinite complex, the Co2 and Co3 metal centres are each coordinated to separate Cl atoms, Cl2 and Cl3, respectively, and are bridged by three diphosphinite ligands *via* their oxygen atoms as shown in Fig. 2 ▸. Moreover, the Co2 and Co3 atoms exhibit a distorted tetra­hedral geometry with O—Co—O and O—Co—Cl bond angles of 103.4 (2)–109.5 (2)° to 108.75 (2)–114.3 (2)°, respectively. The geometric bond parameters around the metal centres in both the cationic and anionic species are comparable with those of closely related structures reported in the literature (Naktode *et al.*, 2014 ▸; Fliedel *et al.*, 2016 ▸).

**Figure 3:**
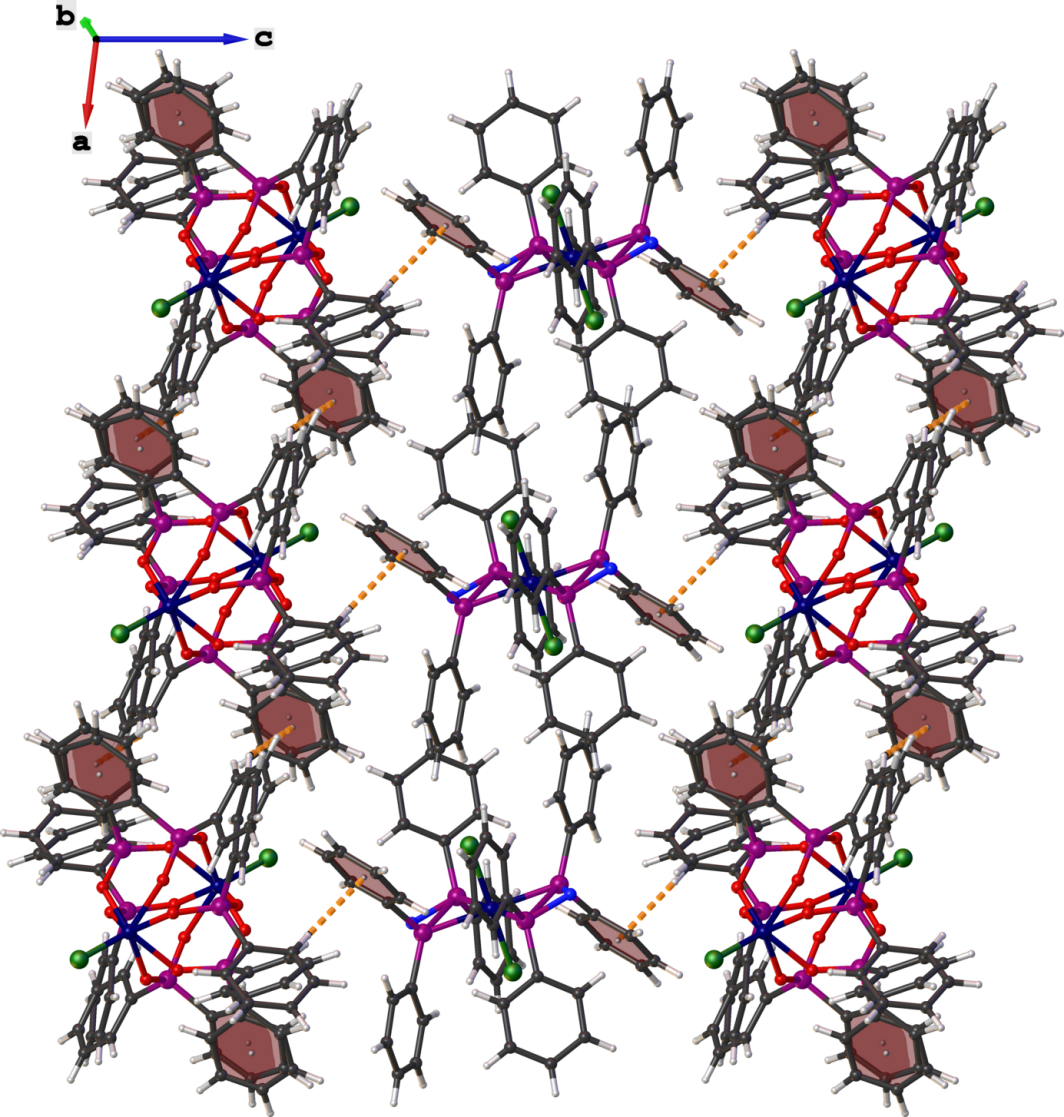
Representation of C—H⋯π hydrogen-bonding patterns in the crystal packing of the title compound.

No π–π stacking inter­actions are observed in the crystal packing of the title compound. However, C—H⋯O hydrogen bonds are found between the H17 atom of the phenyl ring and the O7 atom of the diethyl ether mol­ecule, as well as C—H⋯Cl inter­actions between the H6 atom of the phenyl moiety and Cl4 of the di­chloro­methane mol­ecule (Table 1 ▸). Intra­molecular C—H⋯Cl hydrogen-bonding patterns with a *S*(6) graph-set motif are found between the H2 and Cl1 atoms of the cationic complex (Table 1 ▸). Conversely, inter­molecular C24—H24⋯Cl2 hydrogen bonds link together neighbouring cationic and anionic complexes to form a supra­molecular chain along the crystallographic *c-*axis direction. The crystal packing is further consolidated by inter­molecular C—H⋯π hydrogen bonds between the phenyl rings of neighbouring amino­diphosphine and phosphinite moieties (Table 1 ▸), which form two-dimensional supra­molecular structures along the crystallographic *ac* plane (Fig. 3 ▸).

## Synthesis and crystallization

The title compound was synthesized following a literature procedure (Naicker *et al.*, 2015 ▸). The crude product was recrystallized *via* vapour diffusion of diethyl ether into a di­chloro­methane solution of the crude product to obtain green plate-like single crystals suitable for X-ray diffraction.

## Refinement

Crystallographic data and structure refinement details are summarized in Table 2 ▸. The cyclo­hexa­nyl moiety of the amino­diphosphine ligand exhibits a two-component rotational disorder along the N—C bond, while the di­phenyl­phospho­nate ligands in the anionic complex shows disorder around the inversion centre located between the two Co(II) atoms. The whole anionic species was modelled using PART −1 instruction with 50% site occupancy in the asymmetric unit, while the disordered cyclo­hexa­nyl moiety’s major component was modelled using PART 1 and 2 instructions with 71.1 (7)% site occupancy. The di­chloro­methane and diether ether mol­ecules were modelled using PART 1 and 2 instructions with 59.4 (3)% and 40.6 (3)% site occupancies in the asymmetric unit, respectively.

The geometry of the ether mol­ecule was restrained. The two C—O bond distances were restrained to be similar (s.u. 0.02 Å) and 1,3 distances were restrained to target values of 2.36 (1) for the O—C—C angles, and 2.359 (1) for the C—O—C angle. *U^ij^* components of ADPs for disordered ether and DCM atoms closer to each other than 2.0 Å were restrained to be similar with an s.u. of 0.01 Å^2^. The two disordered cyclo­hexyl moieties were restrained to have similar geometries (SAME restraint with s.u. 0.02 Å. The two N—C bond lengths were restrained to be similar in length (SADI restraint with s.u. 0.02 Angstrom). *U^ij^* components of ADPs for disordered ether and DCM atoms closer to each other than 2.0 Å were restrained to be similar with an s.u. of 0.01 Å^2^. The Co and Cl atoms in the anion are related by pseudo-inversion and the ADPs of cobalt and chlorine atoms were each constrained to be identical (EADP constraint). The Co—Cl bonds as well as all Co—O bonds were each restrained to be similar (SADI restraints with s.u. 0.02 Å). Half of the phenyl rings of the anion (which overlap with their counterparts from inversion) were constrained to resemble ideal hexa­gons with 1.39 A C—C bonds (AFIX 66 constraint). Constrained were C31—C36 and C49—C54.

## Supplementary Material

Crystal structure: contains datablock(s) I. DOI: 10.1107/S2414314625006327/zl4085sup1.cif

Structure factors: contains datablock(s) I. DOI: 10.1107/S2414314625006327/zl4085Isup2.hkl

CCDC reference: 2473254

Additional supporting information:  crystallographic information; 3D view; checkCIF report

## Figures and Tables

**Table 1:** Hydrogen-bond geometry (Å, °) *Cg*1, *Cg*2 and *Cg*3 are the centroids of the C37–C41/C65, C42/C61–C64/C66 and C19–C24 rings, respectively.

*D*—H⋯*A*	*D*—H	H⋯*A*	*D*⋯*A*	*D*—H⋯*A*
C2—H2⋯Cl1^i^	0.95	2.66	3.174 (2)	114
C6—H6⋯Cl4^i^	0.95	2.95	3.668 (4)	133
C17—H17⋯O7	0.95	2.48	3.314 (8)	146
C24—H24⋯Cl2^ii^	0.95	2.96	3.682 (5)	133
C27—H27*B*⋯*Cg*1	0.99	2.97	3.834 (11)	147
C53—H53⋯*Cg*2^iii^	0.95	2.98	3.787 (8)	143
C60—H60⋯*Cg*3^ii^	0.95	2.72	3.626 (8)	159
C29*A*—H29*C*⋯*Cg*2^ii^	0.99	2.69	3.57 (2)	148

Symmetry codes: (i) 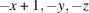; (ii) 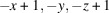; (iii) 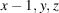.

**Table 2:** Experimental details

Crystal data	
Chemical formula	[CoCl_2_(C_30_H_31_NP_2_)_2_][Co_2_(C_12_H_10_O_2_P)_3_Cl_2_]·1.189CH_2_Cl_2_·0.811C_4_H_10_O
*M*_r_	2066.26
Crystal system, space group	Monoclinic, *P*2_1_/*c*
Temperature (K)	100
*a*, *b*, *c* (Å)	10.365 (6), 23.0516 (5), 19.9984 (6)
β (°)	96.641 (1)
*V* (Å^3^)	4746 (3)
*Z*	2
Radiation type	Cu *K*α
μ (mm^−1^)	7.28
Crystal size (mm)	0.18 × 0.06 × 0.06

Data collection	
Diffractometer	Bruker SMART APEXII area detector
Absorption correction	Multi-scan (*SADABS*; Krause *et al.*, 2015 ▸)
*T*_min_, *T*_max_	0.359, 0.674
No. of measured, independent and observed [*I* > 2σ(*I*)] reflections	18724, 9405, 8330
*R*_int_	0.021
(sin θ/λ)_max_ (Å^−1^)	0.624

Refinement	
*R*[*F*^2^ > 2σ(*F*^2^)], *wR*(*F*^2^), *S*	0.038, 0.093, 1.03
No. of reflections	9405
No. of parameters	848
No. of restraints	297
H-atom treatment	H-atom parameters constrained
Δρ_max_, Δρ_min_ (e Å^−3^)	0.50, −0.49

Computer programs: *COSMO* and *SAINT* (Bruker, 2009 ▸), *SHELXT2018/2* (Sheldrick, 2015*a* ▸), *SHELX2018/3* (Sheldrick, 2015*b* ▸), *OLEX2* (Dolomanov *et al.*, 2009 ▸).

## full crystallographic data

### Bis[N,N-bis(diphenylphosphanyl)cyclohexanamine-κ2P,P']dichloridocobalt(III) tris(µ-diphenylphosphinato-κ2O:O')bis[chloridocobaltate(II)]–dichloromethane–diethyl ether (1/1.189/0.811) Crystal data

**Table:**

[CoCl_2_(C_30_H_31_NP_2_)_2_][Co_2_(C_12_H_10_O_2_P)_3_Cl_2_]·1.189CH_2_Cl_2_·0.811C_4_H_10_O	*F*(000) = 2136
*M**_r_* = 2066.26	*D*_x_ = 1.446 Mg m^−^^3^
Monoclinic, *P*2_1_/*c*	Cu *K*α radiation, λ = 1.54184 Å
*a* = 10.365 (6) Å	Cell parameters from 9277 reflections
*b* = 23.0516 (5) Å	θ = 2.5–28.4°
*c* = 19.9984 (6) Å	µ = 7.28 mm^−^^1^
β = 96.641 (1)°	*T* = 100 K
*V* = 4746 (3) Å^3^	Plate, green
*Z* = 2	0.18 × 0.06 × 0.06 mm

### Bis[N,N-bis(diphenylphosphanyl)cyclohexanamine-κ2P,P']dichloridocobalt(III) tris(µ-diphenylphosphinato-κ2O:O')bis[chloridocobaltate(II)]–dichloromethane–diethyl ether (1/1.189/0.811) Data collection

**Table:**

Bruker SMART APEXII area detector diffractometer	8330 reflections with *I* > 2σ(*I*)
Detector resolution: 7.9 pixels mm^-1^	*R*_int_ = 0.021
ω and φ scans	θ_max_ = 74.2°, θ_min_ = 3.8°
Absorption correction: multi-scan (SADABS; Krause *et al.*, 2015)	*h* = −12→12
*T*_min_ = 0.359, *T*_max_ = 0.674	*k* = −28→25
18724 measured reflections	*l* = −24→20
9405 independent reflections	

### Bis[N,N-bis(diphenylphosphanyl)cyclohexanamine-κ2P,P']dichloridocobalt(III) tris(µ-diphenylphosphinato-κ2O:O')bis[chloridocobaltate(II)]–dichloromethane–diethyl ether (1/1.189/0.811) Refinement

**Table:**

Refinement on *F*^2^	Primary atom site location: dual
Least-squares matrix: full	Hydrogen site location: inferred from neighbouring sites
*R*[*F*^2^ > 2σ(*F*^2^)] = 0.038	H-atom parameters constrained
*wR*(*F*^2^) = 0.093	*w* = 1/[σ^2^(*F*_o_^2^) + (0.0371*P*)^2^ + 3.8871*P*] where *P* = (*F*_o_^2^ + 2*F*_c_^2^)/3
*S* = 1.03	(Δ/σ)_max_ = 0.001
9405 reflections	Δρ_max_ = 0.50 e Å^−^^3^
848 parameters	Δρ_min_ = −0.49 e Å^−^^3^
297 restraints	

### Bis[N,N-bis(diphenylphosphanyl)cyclohexanamine-κ2P,P']dichloridocobalt(III) tris(µ-diphenylphosphinato-κ2O:O')bis[chloridocobaltate(II)]–dichloromethane–diethyl ether (1/1.189/0.811) Special details

**Table:**

Geometry. All esds (except the esd in the dihedral angle between two l.s. planes) are estimated using the full covariance matrix. The cell esds are taken into account individually in the estimation of esds in distances, angles and torsion angles; correlations between esds in cell parameters are only used when they are defined by crystal symmetry. An approximate (isotropic) treatment of cell esds is used for estimating esds involving l.s. planes.

### Bis[N,N-bis(diphenylphosphanyl)cyclohexanamine-κ2P,P']dichloridocobalt(III) tris(µ-diphenylphosphinato-κ2O:O')bis[chloridocobaltate(II)]–dichloromethane–diethyl ether (1/1.189/0.811) Fractional atomic coordinates and isotropic or equivalent isotropic displacement parameters (Å^2^)

**Table:**

	*x*	*y*	*z*	*U*_iso_*/*U*_eq_	Occ. (<1)
Co1	0.500000	0.000000	0.000000	0.01898 (10)	
Cl1	0.69903 (4)	0.01872 (2)	0.04848 (2)	0.02301 (10)	
P1	0.51415 (5)	−0.08972 (2)	0.04709 (3)	0.02132 (11)	
P2	0.42620 (5)	0.00310 (2)	0.10286 (3)	0.02095 (11)	
N1	0.43784 (17)	−0.07018 (8)	0.11469 (9)	0.0233 (4)	
C1	0.4307 (2)	−0.15243 (9)	0.00676 (11)	0.0269 (4)	
C2	0.2955 (2)	−0.15165 (10)	−0.00755 (11)	0.0297 (5)	
H2	0.247538	−0.119799	0.006838	0.036*	
C3	0.2306 (3)	−0.19715 (11)	−0.04272 (12)	0.0364 (6)	
H3	0.138606	−0.196392	−0.051954	0.044*	
C4	0.3000 (3)	−0.24342 (11)	−0.06419 (13)	0.0415 (6)	
H4	0.255504	−0.274252	−0.088527	0.050*	
C5	0.4332 (3)	−0.24503 (11)	−0.05047 (13)	0.0424 (6)	
H5	0.480583	−0.276835	−0.065480	0.051*	
C6	0.4985 (3)	−0.19995 (10)	−0.01453 (12)	0.0338 (5)	
H6	0.590296	−0.201616	−0.004427	0.041*	
C7	0.6792 (2)	−0.11345 (9)	0.07478 (11)	0.0258 (4)	
C8	0.7311 (2)	−0.11461 (10)	0.14218 (12)	0.0313 (5)	
H8	0.677558	−0.105766	0.176304	0.038*	
C9	0.8610 (2)	−0.12868 (11)	0.15962 (14)	0.0381 (6)	
H9	0.895754	−0.129682	0.205689	0.046*	
C10	0.9402 (2)	−0.14127 (11)	0.11022 (15)	0.0404 (6)	
H10	1.028565	−0.151519	0.122410	0.048*	
C11	0.8901 (2)	−0.13883 (11)	0.04320 (14)	0.0387 (6)	
H11	0.944492	−0.146835	0.009217	0.046*	
C12	0.7608 (2)	−0.12477 (10)	0.02543 (12)	0.0319 (5)	
H12	0.727317	−0.122795	−0.020782	0.038*	
C13	0.2620 (2)	0.02661 (9)	0.11248 (10)	0.0239 (4)	
C14	0.1548 (2)	−0.00558 (10)	0.08462 (11)	0.0251 (4)	
H14	0.167735	−0.041048	0.062148	0.030*	
C15	0.0297 (2)	0.01382 (10)	0.08950 (11)	0.0289 (5)	
H15	−0.042443	−0.008866	0.071334	0.035*	
C16	0.0093 (2)	0.06633 (11)	0.12084 (12)	0.0345 (5)	
H16	−0.076537	0.079879	0.123407	0.041*	
C17	0.1147 (2)	0.09870 (11)	0.14825 (13)	0.0355 (5)	
H17	0.101060	0.134652	0.169576	0.043*	
C18	0.2403 (2)	0.07895 (10)	0.14477 (12)	0.0300 (5)	
H18	0.311996	0.101150	0.164488	0.036*	
C19	0.5293 (2)	0.04136 (10)	0.16797 (11)	0.0265 (4)	
C20	0.5585 (3)	0.09922 (11)	0.15560 (12)	0.0350 (5)	
H20	0.520229	0.117613	0.115652	0.042*	
C21	0.6434 (3)	0.13005 (13)	0.20139 (14)	0.0446 (7)	
H21	0.661727	0.169601	0.193065	0.053*	
C22	0.7010 (3)	0.10325 (13)	0.25895 (15)	0.0477 (7)	
H22	0.760697	0.124073	0.289655	0.057*	
C23	0.6720 (3)	0.04630 (13)	0.27193 (14)	0.0440 (7)	
H23	0.710691	0.028238	0.312012	0.053*	
C24	0.5864 (2)	0.01507 (11)	0.22681 (12)	0.0338 (5)	
H24	0.566859	−0.024177	0.236102	0.041*	
C25	0.4157 (5)	−0.10776 (17)	0.1757 (2)	0.0265 (8)	0.711 (7)
H25	0.492899	−0.102460	0.210098	0.032*	0.711 (7)
C26	0.2969 (5)	−0.0881 (2)	0.2078 (3)	0.0319 (10)	0.711 (7)
H26A	0.218794	−0.091440	0.174479	0.038*	0.711 (7)
H26B	0.307164	−0.046779	0.220985	0.038*	0.711 (7)
C27	0.2784 (6)	−0.1249 (3)	0.2702 (3)	0.0363 (12)	0.711 (7)
H27A	0.351404	−0.117656	0.305696	0.044*	0.711 (7)
H27B	0.196991	−0.113205	0.287943	0.044*	0.711 (7)
C28	0.2726 (5)	−0.18899 (19)	0.2531 (2)	0.0419 (10)	0.711 (7)
H28A	0.194786	−0.196935	0.220855	0.050*	0.711 (7)
H28B	0.265411	−0.211827	0.294434	0.050*	0.711 (7)
C29	0.3946 (6)	−0.2074 (3)	0.2222 (3)	0.0397 (13)	0.711 (7)
H29A	0.389416	−0.249188	0.210878	0.048*	0.711 (7)
H29B	0.472228	−0.201188	0.255234	0.048*	0.711 (7)
C30	0.4071 (5)	−0.17186 (19)	0.1585 (2)	0.0332 (10)	0.711 (7)
H30A	0.485875	−0.184003	0.138573	0.040*	0.711 (7)
H30B	0.330734	−0.178993	0.124997	0.040*	0.711 (7)
C25A	0.3675 (11)	−0.1071 (4)	0.1588 (5)	0.0288 (18)	0.289 (7)
H25A	0.284683	−0.119253	0.131790	0.035*	0.289 (7)
C26A	0.4445 (12)	−0.1622 (5)	0.1769 (6)	0.035 (2)	0.289 (7)
H26C	0.523762	−0.152969	0.207634	0.042*	0.289 (7)
H26D	0.471048	−0.180342	0.135785	0.042*	0.289 (7)
C27A	0.3558 (14)	−0.2047 (7)	0.2122 (7)	0.038 (2)	0.289 (7)
H27C	0.276008	−0.213370	0.181612	0.046*	0.289 (7)
H27D	0.402344	−0.241599	0.223137	0.046*	0.289 (7)
C28A	0.3205 (12)	−0.1767 (5)	0.2758 (5)	0.0409 (19)	0.289 (7)
H28C	0.400238	−0.169605	0.307043	0.049*	0.289 (7)
H28D	0.263465	−0.202899	0.298287	0.049*	0.289 (7)
C29A	0.2504 (15)	−0.1190 (7)	0.2588 (8)	0.036 (2)	0.289 (7)
H29C	0.167031	−0.126849	0.230872	0.043*	0.289 (7)
H29D	0.230529	−0.100382	0.301044	0.043*	0.289 (7)
C30A	0.3306 (12)	−0.0776 (5)	0.2213 (6)	0.031 (2)	0.289 (7)
H30C	0.279692	−0.042103	0.208651	0.037*	0.289 (7)
H30D	0.409995	−0.066092	0.250711	0.037*	0.289 (7)
Co2	0.4494 (3)	0.04230 (11)	0.56458 (15)	0.0252 (3)	0.5
Co3	0.5664 (3)	−0.05433 (11)	0.42347 (15)	0.0252 (3)	0.5
Cl2	0.3763 (4)	0.09917 (13)	0.64369 (18)	0.0339 (4)	0.5
Cl3	0.6335 (4)	−0.11390 (13)	0.34402 (18)	0.0339 (4)	0.5
P4	0.72920 (11)	0.04486 (5)	0.50884 (6)	0.0282 (2)	0.5
O2	0.7141 (3)	−0.01107 (14)	0.46919 (17)	0.0315 (7)	0.5
O4	0.6061 (3)	0.07130 (15)	0.52968 (18)	0.0366 (8)	0.5
C42	0.879 (3)	−0.0231 (12)	0.6915 (16)	0.033 (4)	0.5
H42	0.851949	−0.052302	0.720285	0.039*	0.5
C43	0.8018 (5)	0.0997 (2)	0.4607 (3)	0.0322 (12)	0.5
C44	0.7422 (6)	0.1531 (2)	0.4505 (3)	0.0401 (12)	0.5
H44	0.661027	0.160096	0.466667	0.048*	0.5
C45	0.8015 (11)	0.1968 (4)	0.4161 (5)	0.050 (2)	0.5
H45	0.760476	0.233514	0.408900	0.061*	0.5
C46	0.9196 (8)	0.1865 (4)	0.3929 (4)	0.0419 (19)	0.5
H46	0.960792	0.216671	0.370924	0.050*	0.5
C47	0.9783 (7)	0.1332 (3)	0.4012 (3)	0.0427 (13)	0.5
H47	1.058817	0.126525	0.384115	0.051*	0.5
C48	0.9206 (8)	0.0893 (3)	0.4344 (4)	0.0397 (18)	0.5
H48	0.960706	0.052207	0.439395	0.048*	0.5
C61	0.8380 (6)	0.0314 (3)	0.5845 (3)	0.0289 (13)	0.5
C62	0.9576 (7)	0.0596 (3)	0.6001 (4)	0.0329 (13)	0.5
H62	0.986018	0.087484	0.569961	0.039*	0.5
C63	1.0331 (12)	0.0468 (6)	0.6590 (6)	0.043 (3)	0.5
H63	1.116510	0.064043	0.668168	0.052*	0.5
C64	0.990 (3)	0.0093 (11)	0.7053 (11)	0.043 (4)	0.5
H64	1.039318	0.005622	0.748249	0.052*	0.5
C66	0.817 (3)	−0.0089 (14)	0.6352 (15)	0.035 (5)	0.5
H66	0.738772	−0.030255	0.625113	0.042*	0.5
P3	0.32535 (11)	0.03429 (5)	0.41031 (6)	0.0303 (2)	0.5
O1	0.3194 (3)	0.03453 (17)	0.48573 (17)	0.0381 (8)	0.5
O6	0.4343 (3)	0.00087 (15)	0.38402 (16)	0.0344 (7)	0.5
C31	0.3333 (3)	0.10843 (13)	0.38094 (19)	0.0331 (10)	0.5
C32	0.2854 (4)	0.15319 (17)	0.41756 (17)	0.0403 (12)	0.5
H32	0.253976	0.145209	0.459386	0.048*	0.5
C33	0.2834 (5)	0.20962 (14)	0.3930 (2)	0.049 (4)	0.5
H33	0.250617	0.240214	0.418002	0.058*	0.5
C34	0.3294 (5)	0.22130 (14)	0.3318 (3)	0.0479 (15)	0.5
H34	0.327987	0.259866	0.314976	0.057*	0.5
C35	0.3773 (5)	0.1765 (2)	0.2952 (2)	0.0455 (19)	0.5
H35	0.408717	0.184515	0.253333	0.055*	0.5
C36	0.3793 (4)	0.12010 (17)	0.3197 (2)	0.0383 (17)	0.5
H36	0.412077	0.089510	0.294715	0.046*	0.5
C37	0.187 (3)	0.0052 (15)	0.3781 (17)	0.036 (4)	0.5
C38	0.0971 (9)	−0.0264 (4)	0.4134 (4)	0.0493 (18)	0.5
H38	0.121877	−0.032450	0.460079	0.059*	0.5
C39	−0.0217 (10)	−0.0492 (3)	0.3872 (5)	0.0517 (19)	0.5
H39	−0.073444	−0.069306	0.415899	0.062*	0.5
C40	−0.0651 (10)	−0.0431 (6)	0.3202 (6)	0.035 (2)	0.5
H40	−0.142491	−0.061983	0.301732	0.043*	0.5
C41	0.004 (2)	−0.0091 (11)	0.2794 (12)	0.041 (4)	0.5
H41	−0.030050	0.000467	0.234656	0.049*	0.5
C65	0.129 (3)	0.0106 (11)	0.3079 (15)	0.031 (4)	0.5
H65	0.180760	0.029405	0.278028	0.038*	0.5
P5	0.46798 (11)	−0.09748 (5)	0.56283 (6)	0.0266 (2)	0.5
O3	0.4792 (3)	−0.03785 (13)	0.59485 (16)	0.0309 (7)	0.5
O5	0.4873 (3)	−0.10143 (14)	0.48930 (16)	0.0329 (7)	0.5
C49	0.3095 (3)	−0.12660 (16)	0.57327 (18)	0.0302 (10)	0.5
C50	0.2919 (4)	−0.18605 (15)	0.5795 (2)	0.0363 (11)	0.5
H50	0.363768	−0.211605	0.579499	0.044*	0.5
C51	0.1691 (6)	−0.20810 (16)	0.5859 (3)	0.047 (3)	0.5
H51	0.157019	−0.248732	0.590151	0.056*	0.5
C52	0.0639 (4)	−0.1707 (3)	0.5859 (4)	0.047 (2)	0.5
H52	−0.020075	−0.185786	0.590247	0.056*	0.5
C53	0.0815 (3)	−0.1113 (2)	0.5797 (3)	0.043 (2)	0.5
H53	0.009577	−0.085713	0.579691	0.052*	0.5
C54	0.2043 (4)	−0.08921 (14)	0.5733 (2)	0.0341 (12)	0.5
H54	0.216326	−0.048585	0.569039	0.041*	0.5
C55	0.5828 (4)	−0.14520 (19)	0.6097 (2)	0.0290 (9)	0.5
C56	0.6535 (6)	−0.1851 (3)	0.5763 (3)	0.0344 (10)	0.5
H56	0.646478	−0.185148	0.528519	0.041*	0.5
C57	0.7337 (11)	−0.2247 (5)	0.6126 (5)	0.0400 (17)	0.5
H57	0.781222	−0.251905	0.589556	0.048*	0.5
C58	0.7453 (6)	−0.2249 (2)	0.6816 (3)	0.0404 (13)	0.5
H58	0.799933	−0.252543	0.706061	0.048*	0.5
C59	0.6776 (6)	−0.1851 (3)	0.7156 (4)	0.0407 (17)	0.5
H59	0.686093	−0.185287	0.763413	0.049*	0.5
C60	0.5967 (7)	−0.1444 (3)	0.6798 (3)	0.0351 (15)	0.5
H60	0.551617	−0.116389	0.703122	0.042*	0.5
Cl4	0.2005 (4)	0.24516 (9)	0.07969 (18)	0.1402 (15)	0.595 (3)
Cl5	0.01892 (14)	0.28376 (9)	0.16786 (9)	0.0713 (6)	0.595 (3)
C71	0.1010 (7)	0.3014 (3)	0.0968 (4)	0.0688 (16)	0.595 (3)
H71A	0.036610	0.308867	0.057232	0.083*	0.595 (3)
H71B	0.153258	0.337014	0.106319	0.083*	0.595 (3)
O7	0.0675 (8)	0.2407 (3)	0.1545 (3)	0.0861 (19)	0.405 (3)
C67	−0.0431 (14)	0.2098 (6)	0.2436 (6)	0.125 (5)	0.405 (3)
H67A	−0.070851	0.224026	0.285847	0.187*	0.405 (3)
H67B	0.018974	0.177939	0.253240	0.187*	0.405 (3)
H67C	−0.118806	0.195782	0.214166	0.187*	0.405 (3)
C68	0.0181 (10)	0.2563 (4)	0.2106 (5)	0.073 (2)	0.405 (3)
H68A	−0.046674	0.287401	0.199289	0.088*	0.405 (3)
H68B	0.088741	0.272765	0.242583	0.088*	0.405 (3)
C69	0.1534 (13)	0.2818 (6)	0.1287 (7)	0.101 (3)	0.405 (3)
H69A	0.244715	0.275492	0.147897	0.122*	0.405 (3)
H69B	0.127937	0.322232	0.137501	0.122*	0.405 (3)
C70	0.1330 (12)	0.2676 (6)	0.0540 (4)	0.081 (3)	0.405 (3)
H70A	0.041657	0.273926	0.036715	0.122*	0.405 (3)
H70B	0.155837	0.226925	0.047214	0.122*	0.405 (3)
H70C	0.188166	0.292734	0.029849	0.122*	0.405 (3)

### Bis[N,N-bis(diphenylphosphanyl)cyclohexanamine-κ2P,P']dichloridocobalt(III) tris(µ-diphenylphosphinato-κ2O:O')bis[chloridocobaltate(II)]–dichloromethane–diethyl ether (1/1.189/0.811) Atomic displacement parameters (Å^2^)

**Table:**

	*U*^11^	*U*^22^	*U*^33^	*U*^12^	*U*^13^	*U*^23^
Co1	0.0164 (2)	0.0190 (2)	0.0205 (2)	−0.00092 (17)	−0.00225 (17)	−0.00228 (17)
Cl1	0.0173 (2)	0.0249 (2)	0.0254 (2)	−0.00158 (18)	−0.00343 (17)	−0.00280 (18)
P1	0.0215 (2)	0.0196 (2)	0.0217 (2)	0.00004 (19)	−0.00208 (19)	−0.00153 (19)
P2	0.0189 (2)	0.0220 (2)	0.0211 (2)	−0.00144 (19)	−0.00101 (18)	−0.00325 (19)
N1	0.0235 (9)	0.0225 (9)	0.0233 (9)	−0.0007 (7)	−0.0006 (7)	0.0002 (7)
C1	0.0360 (12)	0.0215 (10)	0.0221 (10)	−0.0032 (9)	−0.0011 (9)	−0.0006 (8)
C2	0.0354 (12)	0.0256 (11)	0.0274 (11)	−0.0064 (9)	0.0003 (9)	0.0003 (9)
C3	0.0402 (14)	0.0363 (13)	0.0310 (12)	−0.0134 (11)	−0.0033 (10)	−0.0020 (10)
C4	0.0588 (17)	0.0314 (13)	0.0318 (13)	−0.0146 (12)	−0.0059 (11)	−0.0052 (10)
C5	0.0598 (18)	0.0254 (12)	0.0401 (14)	−0.0009 (12)	−0.0025 (12)	−0.0078 (10)
C6	0.0408 (14)	0.0246 (11)	0.0341 (12)	0.0016 (10)	−0.0040 (10)	−0.0032 (9)
C7	0.0249 (11)	0.0181 (10)	0.0327 (11)	0.0034 (8)	−0.0031 (8)	0.0012 (8)
C8	0.0327 (12)	0.0255 (11)	0.0337 (12)	0.0006 (9)	−0.0049 (9)	−0.0023 (9)
C9	0.0349 (13)	0.0320 (12)	0.0431 (14)	0.0037 (10)	−0.0135 (11)	−0.0015 (10)
C10	0.0252 (12)	0.0303 (13)	0.0634 (17)	0.0057 (10)	−0.0049 (11)	0.0015 (12)
C11	0.0332 (13)	0.0297 (12)	0.0543 (16)	0.0067 (10)	0.0096 (11)	0.0031 (11)
C12	0.0341 (12)	0.0268 (11)	0.0343 (12)	0.0069 (9)	0.0026 (10)	0.0035 (9)
C13	0.0230 (10)	0.0251 (10)	0.0229 (10)	0.0007 (8)	−0.0001 (8)	−0.0002 (8)
C14	0.0241 (11)	0.0273 (11)	0.0232 (10)	−0.0001 (8)	0.0000 (8)	−0.0002 (8)
C15	0.0211 (10)	0.0353 (12)	0.0293 (11)	0.0013 (9)	−0.0015 (8)	0.0007 (9)
C16	0.0256 (12)	0.0419 (14)	0.0354 (13)	0.0091 (10)	0.0014 (9)	0.0002 (10)
C17	0.0345 (13)	0.0329 (12)	0.0383 (13)	0.0095 (10)	0.0008 (10)	−0.0068 (10)
C18	0.0296 (12)	0.0285 (11)	0.0312 (12)	−0.0005 (9)	0.0001 (9)	−0.0047 (9)
C19	0.0218 (10)	0.0313 (11)	0.0265 (11)	−0.0028 (9)	0.0028 (8)	−0.0094 (9)
C20	0.0413 (14)	0.0345 (13)	0.0293 (12)	−0.0102 (11)	0.0045 (10)	−0.0091 (10)
C21	0.0493 (16)	0.0413 (15)	0.0439 (15)	−0.0196 (13)	0.0093 (12)	−0.0188 (12)
C22	0.0356 (14)	0.0545 (17)	0.0503 (16)	−0.0089 (12)	−0.0070 (12)	−0.0288 (14)
C23	0.0349 (14)	0.0486 (16)	0.0439 (15)	0.0063 (12)	−0.0149 (11)	−0.0192 (12)
C24	0.0302 (12)	0.0339 (12)	0.0348 (12)	0.0043 (10)	−0.0073 (9)	−0.0102 (10)
C25	0.030 (2)	0.0258 (16)	0.0225 (18)	−0.0020 (16)	−0.0022 (15)	0.0028 (13)
C26	0.033 (2)	0.033 (2)	0.029 (2)	−0.0019 (17)	0.0020 (17)	0.0033 (16)
C27	0.037 (3)	0.045 (2)	0.027 (2)	−0.0009 (19)	0.0049 (19)	0.0095 (17)
C28	0.054 (3)	0.041 (2)	0.030 (2)	−0.0106 (18)	0.0035 (18)	0.0116 (16)
C29	0.056 (3)	0.0320 (19)	0.030 (2)	−0.001 (2)	0.003 (2)	0.0074 (17)
C30	0.045 (2)	0.0263 (19)	0.027 (2)	−0.0036 (16)	0.0000 (16)	0.0030 (15)
C25A	0.029 (4)	0.031 (3)	0.025 (4)	−0.003 (3)	−0.002 (3)	0.006 (3)
C26A	0.044 (4)	0.032 (4)	0.029 (4)	−0.001 (3)	0.003 (3)	0.003 (3)
C27A	0.050 (5)	0.032 (4)	0.032 (4)	−0.007 (4)	0.000 (4)	0.008 (3)
C28A	0.049 (4)	0.041 (4)	0.032 (4)	−0.009 (3)	0.002 (3)	0.010 (3)
C29A	0.040 (5)	0.042 (4)	0.026 (4)	−0.003 (4)	0.000 (4)	0.005 (3)
C30A	0.033 (4)	0.034 (4)	0.025 (4)	−0.001 (3)	0.001 (3)	0.004 (3)
Co2	0.0222 (6)	0.0238 (9)	0.0288 (9)	0.0017 (6)	−0.0004 (5)	−0.0018 (6)
Co3	0.0222 (6)	0.0238 (9)	0.0288 (9)	0.0017 (6)	−0.0004 (5)	−0.0018 (6)
Cl2	0.0334 (6)	0.0350 (14)	0.0327 (12)	0.0061 (9)	0.0014 (7)	−0.0048 (8)
Cl3	0.0334 (6)	0.0350 (14)	0.0327 (12)	0.0061 (9)	0.0014 (7)	−0.0048 (8)
P4	0.0247 (5)	0.0247 (5)	0.0343 (6)	0.0002 (4)	−0.0003 (4)	−0.0019 (4)
O2	0.0297 (17)	0.0246 (16)	0.0387 (19)	−0.0031 (13)	−0.0015 (14)	−0.0026 (14)
O4	0.0271 (17)	0.0383 (19)	0.045 (2)	0.0057 (15)	0.0063 (14)	0.0046 (15)
C42	0.027 (4)	0.028 (9)	0.042 (6)	0.005 (6)	−0.003 (3)	0.003 (6)
C43	0.033 (3)	0.023 (2)	0.039 (3)	−0.0005 (19)	−0.001 (2)	−0.006 (2)
C44	0.045 (3)	0.027 (3)	0.048 (3)	0.009 (3)	0.006 (2)	0.000 (3)
C45	0.081 (8)	0.024 (3)	0.044 (4)	0.002 (4)	0.001 (4)	−0.001 (3)
C46	0.058 (4)	0.029 (3)	0.037 (4)	−0.012 (3)	0.002 (3)	0.003 (3)
C47	0.043 (4)	0.035 (3)	0.050 (3)	−0.007 (3)	0.007 (3)	0.000 (3)
C48	0.042 (4)	0.033 (4)	0.044 (4)	0.002 (3)	0.004 (3)	−0.004 (3)
C61	0.018 (3)	0.032 (3)	0.035 (3)	0.001 (3)	0.002 (3)	−0.007 (2)
C62	0.024 (3)	0.034 (3)	0.040 (4)	−0.001 (3)	−0.001 (3)	0.000 (2)
C63	0.021 (5)	0.053 (5)	0.051 (9)	−0.010 (4)	−0.008 (4)	−0.013 (6)
C64	0.037 (6)	0.055 (6)	0.034 (10)	0.014 (5)	−0.010 (5)	−0.002 (5)
C66	0.052 (7)	0.028 (5)	0.022 (12)	−0.001 (5)	−0.004 (6)	0.003 (6)
P3	0.0244 (6)	0.0325 (6)	0.0324 (6)	0.0052 (5)	−0.0033 (4)	−0.0029 (5)
O1	0.0279 (17)	0.051 (2)	0.0337 (19)	0.0033 (16)	−0.0056 (14)	−0.0054 (16)
O6	0.0308 (18)	0.0386 (19)	0.0330 (17)	0.0078 (14)	0.0003 (14)	−0.0001 (14)
C31	0.020 (2)	0.033 (2)	0.044 (3)	0.0022 (19)	−0.0052 (19)	−0.010 (2)
C32	0.028 (3)	0.037 (3)	0.055 (3)	−0.001 (2)	−0.002 (2)	−0.017 (2)
C33	0.034 (5)	0.037 (6)	0.075 (7)	−0.001 (4)	0.006 (5)	−0.021 (5)
C34	0.034 (3)	0.028 (3)	0.081 (5)	−0.007 (3)	0.004 (3)	−0.004 (3)
C35	0.049 (5)	0.034 (4)	0.055 (5)	−0.006 (4)	0.010 (4)	−0.008 (4)
C36	0.031 (3)	0.035 (4)	0.048 (3)	−0.002 (3)	−0.001 (2)	−0.011 (3)
C37	0.059 (6)	0.031 (5)	0.015 (9)	0.016 (4)	−0.011 (5)	0.004 (5)
C38	0.051 (5)	0.054 (5)	0.038 (3)	−0.022 (5)	−0.013 (4)	0.020 (3)
C39	0.056 (6)	0.051 (4)	0.044 (5)	−0.021 (4)	−0.012 (5)	0.014 (4)
C40	0.028 (6)	0.038 (4)	0.039 (6)	−0.010 (4)	−0.003 (4)	−0.011 (4)
C41	0.032 (5)	0.056 (5)	0.033 (9)	−0.012 (4)	−0.007 (5)	0.001 (5)
C65	0.036 (6)	0.029 (10)	0.031 (5)	0.004 (5)	0.008 (4)	−0.001 (6)
P5	0.0263 (5)	0.0251 (5)	0.0282 (6)	−0.0016 (4)	0.0016 (4)	−0.0017 (4)
O3	0.0331 (17)	0.0260 (16)	0.0325 (17)	−0.0002 (13)	−0.0006 (13)	−0.0024 (13)
O5	0.0366 (18)	0.0296 (17)	0.0326 (17)	−0.0058 (14)	0.0049 (14)	−0.0022 (13)
C49	0.032 (2)	0.033 (3)	0.025 (2)	−0.006 (2)	−0.0002 (18)	−0.004 (2)
C50	0.037 (3)	0.031 (3)	0.040 (3)	−0.009 (3)	0.004 (2)	−0.001 (2)
C51	0.037 (4)	0.042 (5)	0.062 (5)	−0.011 (3)	0.009 (3)	−0.005 (4)
C52	0.031 (3)	0.061 (7)	0.048 (5)	−0.012 (4)	0.003 (3)	0.002 (4)
C53	0.027 (3)	0.058 (7)	0.045 (4)	0.009 (4)	0.000 (3)	−0.007 (4)
C54	0.031 (3)	0.037 (3)	0.033 (3)	−0.002 (2)	−0.001 (2)	−0.002 (2)
C55	0.025 (2)	0.023 (2)	0.038 (2)	−0.0041 (18)	0.0008 (18)	−0.0035 (18)
C56	0.033 (3)	0.032 (3)	0.038 (3)	−0.001 (3)	0.003 (2)	−0.009 (2)
C57	0.034 (3)	0.030 (4)	0.054 (4)	0.002 (3)	−0.004 (3)	−0.010 (3)
C58	0.042 (3)	0.024 (2)	0.052 (3)	0.003 (2)	−0.012 (3)	−0.008 (2)
C59	0.049 (4)	0.030 (3)	0.040 (3)	0.005 (3)	−0.010 (3)	−0.003 (2)
C60	0.039 (4)	0.031 (4)	0.034 (3)	0.004 (3)	0.000 (2)	−0.008 (3)
Cl4	0.213 (3)	0.0556 (11)	0.183 (3)	0.0045 (15)	0.152 (3)	−0.0170 (14)
Cl5	0.0467 (8)	0.0986 (14)	0.0709 (10)	−0.0055 (8)	0.0158 (7)	−0.0365 (9)
C71	0.063 (4)	0.063 (4)	0.080 (4)	−0.007 (3)	0.008 (3)	0.000 (3)
O7	0.106 (4)	0.056 (3)	0.104 (4)	−0.021 (3)	0.040 (4)	−0.002 (3)
C67	0.141 (11)	0.137 (11)	0.109 (9)	0.055 (9)	0.068 (8)	0.020 (8)
C68	0.084 (6)	0.043 (4)	0.095 (6)	0.016 (4)	0.019 (5)	−0.013 (4)
C69	0.102 (6)	0.085 (5)	0.122 (6)	−0.019 (5)	0.033 (5)	0.002 (5)
C70	0.082 (6)	0.123 (8)	0.042 (4)	−0.079 (6)	0.017 (4)	−0.022 (5)

### Bis[N,N-bis(diphenylphosphanyl)cyclohexanamine-κ2P,P']dichloridocobalt(III) tris(µ-diphenylphosphinato-κ2O:O')bis[chloridocobaltate(II)]–dichloromethane–diethyl ether (1/1.189/0.811) Geometric parameters (Å, º)

**Table:**

Co1—Cl1^i^	2.2179 (11)	Co3—O6	1.966 (4)
Co1—Cl1	2.2179 (11)	Co3—O5	1.958 (4)
Co1—P1^i^	2.2701 (5)	P4—O2	1.512 (3)
Co1—P1	2.2701 (5)	P4—O4	1.516 (3)
Co1—P2^i^	2.2770 (6)	P4—C43	1.805 (6)
Co1—P2	2.2771 (6)	P4—C61	1.807 (6)
P1—P2	2.6243 (8)	C42—H42	0.9500
P1—N1	1.7038 (19)	C42—C64	1.37 (5)
P1—C1	1.824 (2)	C42—C66	1.27 (4)
P1—C7	1.820 (2)	C43—C44	1.382 (7)
P2—N1	1.7080 (18)	C43—C48	1.415 (10)
P2—C13	1.817 (2)	C44—H44	0.9500
P2—C19	1.815 (2)	C44—C45	1.402 (13)
N1—C25	1.536 (4)	C45—H45	0.9500
N1—C25A	1.478 (9)	C45—C46	1.379 (13)
C1—C2	1.398 (3)	C46—H46	0.9500
C1—C6	1.394 (3)	C46—C47	1.372 (10)
C2—H2	0.9500	C47—H47	0.9500
C2—C3	1.392 (3)	C47—C48	1.385 (9)
C3—H3	0.9500	C48—H48	0.9500
C3—C4	1.382 (4)	C61—C62	1.402 (8)
C4—H4	0.9500	C61—C66	1.41 (3)
C4—C5	1.378 (4)	C62—H62	0.9500
C5—H5	0.9500	C62—C63	1.370 (10)
C5—C6	1.393 (3)	C63—H63	0.9500
C6—H6	0.9500	C63—C64	1.38 (3)
C7—C8	1.393 (3)	C64—H64	0.9500
C7—C12	1.397 (3)	C66—H66	0.9500
C8—H8	0.9500	P3—O1	1.517 (4)
C8—C9	1.390 (3)	P3—O6	1.511 (3)
C9—H9	0.9500	P3—C31	1.812 (3)
C9—C10	1.386 (4)	P3—C37	1.65 (3)
C10—H10	0.9500	C31—C32	1.3900
C10—C11	1.381 (4)	C31—C36	1.3900
C11—H11	0.9500	C32—H32	0.9500
C11—C12	1.384 (3)	C32—C33	1.3900
C12—H12	0.9500	C33—H33	0.9500
C13—C14	1.398 (3)	C33—C34	1.3900
C13—C18	1.399 (3)	C34—H34	0.9500
C14—H14	0.9500	C34—C35	1.3900
C14—C15	1.385 (3)	C35—H35	0.9500
C15—H15	0.9500	C35—C36	1.3900
C15—C16	1.391 (3)	C36—H36	0.9500
C16—H16	0.9500	C37—C38	1.43 (3)
C16—C17	1.382 (4)	C37—C65	1.47 (4)
C17—H17	0.9500	C38—H38	0.9500
C17—C18	1.388 (3)	C38—C39	1.385 (10)
C18—H18	0.9500	C39—H39	0.9500
C19—C20	1.396 (3)	C39—C40	1.370 (10)
C19—C24	1.393 (3)	C40—H40	0.9500
C20—H20	0.9500	C40—C41	1.39 (3)
C20—C21	1.389 (3)	C41—H41	0.9500
C21—H21	0.9500	C41—C65	1.43 (5)
C21—C22	1.380 (4)	C65—H65	0.9500
C22—H22	0.9500	P5—O3	1.515 (3)
C22—C23	1.378 (4)	P5—O5	1.510 (3)
C23—H23	0.9500	P5—C49	1.809 (3)
C23—C24	1.391 (3)	P5—C55	1.801 (5)
C24—H24	0.9500	C49—C50	1.3900
C25—H25	1.0000	C49—C54	1.3900
C25—C26	1.521 (7)	C50—H50	0.9500
C25—C30	1.518 (6)	C50—C51	1.3900
C26—H26A	0.9900	C51—H51	0.9500
C26—H26B	0.9900	C51—C52	1.3900
C26—C27	1.540 (7)	C52—H52	0.9500
C27—H27A	0.9900	C52—C53	1.3900
C27—H27B	0.9900	C53—H53	0.9500
C27—C28	1.516 (8)	C53—C54	1.3900
C28—H28A	0.9900	C54—H54	0.9500
C28—H28B	0.9900	C55—C56	1.393 (7)
C28—C29	1.530 (8)	C55—C60	1.393 (8)
C29—H29A	0.9900	C56—H56	0.9500
C29—H29B	0.9900	C56—C57	1.382 (12)
C29—C30	1.533 (7)	C57—H57	0.9500
C30—H30A	0.9900	C57—C58	1.371 (11)
C30—H30B	0.9900	C58—H58	0.9500
C25A—H25A	1.0000	C58—C59	1.381 (9)
C25A—C26A	1.520 (14)	C59—H59	0.9500
C25A—C30A	1.511 (15)	C59—C60	1.399 (9)
C26A—H26C	0.9900	C60—H60	0.9500
C26A—H26D	0.9900	Cl4—C71	1.715 (7)
C26A—C27A	1.566 (16)	Cl5—C71	1.785 (7)
C27A—H27C	0.9900	C71—H71A	0.9900
C27A—H27D	0.9900	C71—H71B	0.9900
C27A—C28A	1.509 (16)	O7—C68	1.335 (9)
C28A—H28C	0.9900	O7—C69	1.436 (9)
C28A—H28D	0.9900	C67—H67A	0.9800
C28A—C29A	1.533 (15)	C67—H67B	0.9800
C29A—H29C	0.9900	C67—H67C	0.9800
C29A—H29D	0.9900	C67—C68	1.445 (14)
C29A—C30A	1.520 (17)	C68—H68A	0.9900
C30A—H30C	0.9900	C68—H68B	0.9900
C30A—H30D	0.9900	C69—H69A	0.9900
Co2—Cl2	2.252 (2)	C69—H69B	0.9900
Co2—O4	1.958 (5)	C69—C70	1.520 (15)
Co2—O1	1.960 (4)	C70—H70A	0.9800
Co2—O3	1.958 (4)	C70—H70B	0.9800
Co3—Cl3	2.269 (2)	C70—H70C	0.9800
Co3—O2	1.962 (4)		
			
Cl1^i^—Co1—Cl1	180.00 (3)	C25A—C30A—H30D	109.8
Cl1—Co1—P1	89.065 (18)	C29A—C30A—H30C	109.8
Cl1^i^—Co1—P1	90.935 (18)	C29A—C30A—H30D	109.8
Cl1—Co1—P1^i^	90.935 (18)	H30C—C30A—H30D	108.3
Cl1^i^—Co1—P1^i^	89.065 (18)	O4—Co2—Cl2	114.3 (2)
Cl1—Co1—P2^i^	90.48 (3)	O4—Co2—O1	105.4 (2)
Cl1^i^—Co1—P2^i^	89.52 (3)	O1—Co2—Cl2	111.26 (19)
Cl1^i^—Co1—P2	90.48 (3)	O3—Co2—Cl2	112.7 (2)
Cl1—Co1—P2	89.52 (3)	O3—Co2—O4	108.94 (18)
P1^i^—Co1—P1	180.0	O3—Co2—O1	103.41 (19)
P1^i^—Co1—P2	109.501 (19)	O2—Co3—Cl3	110.34 (19)
P1^i^—Co1—P2^i^	70.500 (19)	O2—Co3—O6	109.12 (19)
P1—Co1—P2	70.497 (19)	O6—Co3—Cl3	111.5 (2)
P1—Co1—P2^i^	109.502 (19)	O5—Co3—Cl3	108.75 (19)
P2^i^—Co1—P2	180.0	O5—Co3—O2	109.5 (2)
Co1—P1—P2	54.876 (16)	O5—Co3—O6	107.60 (19)
N1—P1—Co1	94.29 (6)	O2—P4—O4	116.6 (2)
N1—P1—P2	39.78 (6)	O2—P4—C43	110.0 (2)
N1—P1—C1	108.69 (10)	O2—P4—C61	108.1 (2)
N1—P1—C7	110.14 (10)	O4—P4—C43	106.4 (2)
C1—P1—Co1	122.28 (7)	O4—P4—C61	107.6 (2)
C1—P1—P2	131.60 (8)	C43—P4—C61	108.0 (3)
C7—P1—Co1	114.42 (7)	P4—O2—Co3	134.9 (2)
C7—P1—P2	118.44 (7)	P4—O4—Co2	136.1 (2)
C7—P1—C1	106.08 (10)	C64—C42—H42	124.3
Co1—P2—P1	54.627 (17)	C66—C42—H42	124.3
N1—P2—Co1	93.92 (6)	C66—C42—C64	111 (3)
N1—P2—P1	39.66 (6)	C44—C43—P4	119.7 (5)
N1—P2—C13	109.41 (10)	C44—C43—C48	119.3 (6)
N1—P2—C19	110.79 (10)	C48—C43—P4	121.0 (4)
C13—P2—Co1	121.43 (7)	C43—C44—H44	120.0
C13—P2—P1	131.59 (7)	C43—C44—C45	120.0 (6)
C19—P2—Co1	115.59 (8)	C45—C44—H44	120.0
C19—P2—P1	119.77 (8)	C44—C45—H45	120.1
C19—P2—C13	105.07 (10)	C46—C45—C44	119.8 (7)
P1—N1—P2	100.56 (10)	C46—C45—H45	120.1
C25—N1—P1	127.40 (19)	C45—C46—H46	119.6
C25—N1—P2	130.79 (19)	C47—C46—C45	120.8 (8)
C25A—N1—P1	128.9 (4)	C47—C46—H46	119.6
C25A—N1—P2	128.4 (4)	C46—C47—H47	119.9
C2—C1—P1	119.55 (17)	C46—C47—C48	120.2 (6)
C6—C1—P1	121.86 (19)	C48—C47—H47	119.9
C6—C1—C2	118.4 (2)	C43—C48—H48	120.1
C1—C2—H2	119.7	C47—C48—C43	119.8 (6)
C3—C2—C1	120.6 (2)	C47—C48—H48	120.1
C3—C2—H2	119.7	C62—C61—P4	123.8 (5)
C2—C3—H3	120.0	C62—C61—C66	110.6 (12)
C4—C3—C2	120.0 (2)	C66—C61—P4	125.6 (12)
C4—C3—H3	120.0	C61—C62—H62	120.2
C3—C4—H4	119.8	C63—C62—C61	119.5 (8)
C5—C4—C3	120.3 (2)	C63—C62—H62	120.2
C5—C4—H4	119.8	C62—C63—H63	119.4
C4—C5—H5	120.0	C62—C63—C64	121.2 (14)
C4—C5—C6	119.9 (3)	C64—C63—H63	119.4
C6—C5—H5	120.0	C42—C64—C63	122 (2)
C1—C6—H6	119.6	C42—C64—H64	118.9
C5—C6—C1	120.8 (2)	C63—C64—H64	118.9
C5—C6—H6	119.6	C42—C66—C61	134 (3)
C8—C7—P1	122.99 (18)	C42—C66—H66	112.8
C8—C7—C12	118.8 (2)	C61—C66—H66	112.8
C12—C7—P1	117.78 (17)	O1—P3—C31	109.1 (2)
C7—C8—H8	119.9	O1—P3—C37	104.9 (12)
C9—C8—C7	120.1 (2)	O6—P3—O1	117.8 (2)
C9—C8—H8	119.9	O6—P3—C31	107.70 (19)
C8—C9—H9	119.8	O6—P3—C37	108.0 (13)
C10—C9—C8	120.4 (2)	C37—P3—C31	109.2 (12)
C10—C9—H9	119.8	P3—O1—Co2	134.2 (2)
C9—C10—H10	120.1	P3—O6—Co3	134.8 (2)
C11—C10—C9	119.7 (2)	C32—C31—P3	119.8 (2)
C11—C10—H10	120.1	C32—C31—C36	120.0
C10—C11—H11	119.9	C36—C31—P3	120.1 (2)
C10—C11—C12	120.2 (2)	C31—C32—H32	120.0
C12—C11—H11	119.9	C33—C32—C31	120.0
C7—C12—H12	119.7	C33—C32—H32	120.0
C11—C12—C7	120.7 (2)	C32—C33—H33	120.0
C11—C12—H12	119.7	C34—C33—C32	120.0
C14—C13—P2	120.72 (17)	C34—C33—H33	120.0
C14—C13—C18	118.6 (2)	C33—C34—H34	120.0
C18—C13—P2	120.61 (17)	C33—C34—C35	120.0
C13—C14—H14	119.7	C35—C34—H34	120.0
C15—C14—C13	120.5 (2)	C34—C35—H35	120.0
C15—C14—H14	119.7	C36—C35—C34	120.0
C14—C15—H15	119.8	C36—C35—H35	120.0
C14—C15—C16	120.4 (2)	C31—C36—H36	120.0
C16—C15—H15	119.8	C35—C36—C31	120.0
C15—C16—H16	120.2	C35—C36—H36	120.0
C17—C16—C15	119.6 (2)	C38—C37—P3	127 (2)
C17—C16—H16	120.2	C38—C37—C65	108 (2)
C16—C17—H17	119.8	C65—C37—P3	125 (2)
C16—C17—C18	120.3 (2)	C37—C38—H38	116.3
C18—C17—H17	119.8	C39—C38—C37	127.4 (14)
C13—C18—H18	119.7	C39—C38—H38	116.3
C17—C18—C13	120.5 (2)	C38—C39—H39	119.8
C17—C18—H18	119.7	C40—C39—C38	120.5 (8)
C20—C19—P2	117.33 (18)	C40—C39—H39	119.8
C24—C19—P2	123.50 (18)	C39—C40—H40	120.0
C24—C19—C20	119.0 (2)	C39—C40—C41	120.0 (13)
C19—C20—H20	119.8	C41—C40—H40	120.0
C21—C20—C19	120.4 (2)	C40—C41—H41	121.6
C21—C20—H20	119.8	C40—C41—C65	117 (2)
C20—C21—H21	120.0	C65—C41—H41	121.6
C22—C21—C20	120.0 (3)	C37—C65—H65	116.4
C22—C21—H21	120.0	C41—C65—C37	127 (2)
C21—C22—H22	120.0	C41—C65—H65	116.4
C23—C22—C21	120.0 (2)	O3—P5—C49	108.33 (19)
C23—C22—H22	120.0	O3—P5—C55	108.69 (19)
C22—C23—H23	119.7	O5—P5—O3	117.00 (19)
C22—C23—C24	120.5 (3)	O5—P5—C49	108.31 (18)
C24—C23—H23	119.7	O5—P5—C55	108.4 (2)
C19—C24—H24	120.0	C55—P5—C49	105.51 (19)
C23—C24—C19	120.0 (2)	P5—O3—Co2	136.4 (2)
C23—C24—H24	120.0	P5—O5—Co3	137.3 (2)
N1—C25—H25	107.2	C50—C49—P5	120.4 (2)
C26—C25—N1	111.6 (3)	C50—C49—C54	120.0
C26—C25—H25	107.2	C54—C49—P5	119.5 (2)
C30—C25—N1	112.3 (3)	C49—C50—H50	120.0
C30—C25—H25	107.2	C49—C50—C51	120.0
C30—C25—C26	110.9 (4)	C51—C50—H50	120.0
C25—C26—H26A	109.4	C50—C51—H51	120.0
C25—C26—H26B	109.4	C52—C51—C50	120.0
C25—C26—C27	111.1 (4)	C52—C51—H51	120.0
H26A—C26—H26B	108.0	C51—C52—H52	120.0
C27—C26—H26A	109.4	C53—C52—C51	120.0
C27—C26—H26B	109.4	C53—C52—H52	120.0
C26—C27—H27A	109.4	C52—C53—H53	120.0
C26—C27—H27B	109.4	C52—C53—C54	120.0
H27A—C27—H27B	108.0	C54—C53—H53	120.0
C28—C27—C26	111.1 (5)	C49—C54—H54	120.0
C28—C27—H27A	109.4	C53—C54—C49	120.0
C28—C27—H27B	109.4	C53—C54—H54	120.0
C27—C28—H28A	109.6	C56—C55—P5	120.4 (4)
C27—C28—H28B	109.6	C60—C55—P5	120.0 (4)
C27—C28—C29	110.4 (5)	C60—C55—C56	119.5 (5)
H28A—C28—H28B	108.1	C55—C56—H56	119.9
C29—C28—H28A	109.6	C57—C56—C55	120.1 (6)
C29—C28—H28B	109.6	C57—C56—H56	119.9
C28—C29—H29A	109.7	C56—C57—H57	119.7
C28—C29—H29B	109.7	C58—C57—C56	120.6 (9)
C28—C29—C30	109.9 (5)	C58—C57—H57	119.7
H29A—C29—H29B	108.2	C57—C58—H58	119.9
C30—C29—H29A	109.7	C57—C58—C59	120.2 (8)
C30—C29—H29B	109.7	C59—C58—H58	119.9
C25—C30—C29	109.9 (4)	C58—C59—H59	119.9
C25—C30—H30A	109.7	C58—C59—C60	120.1 (7)
C25—C30—H30B	109.7	C60—C59—H59	119.9
C29—C30—H30A	109.7	C55—C60—C59	119.5 (6)
C29—C30—H30B	109.7	C55—C60—H60	120.3
H30A—C30—H30B	108.2	C59—C60—H60	120.3
N1—C25A—H25A	106.8	Cl4—C71—Cl5	109.9 (4)
N1—C25A—C26A	110.0 (8)	Cl4—C71—H71A	109.7
N1—C25A—C30A	115.0 (8)	Cl4—C71—H71B	109.7
C26A—C25A—H25A	106.8	Cl5—C71—H71A	109.7
C30A—C25A—H25A	106.8	Cl5—C71—H71B	109.7
C30A—C25A—C26A	110.8 (10)	H71A—C71—H71B	108.2
C25A—C26A—H26C	110.1	C68—O7—C69	116.0 (8)
C25A—C26A—H26D	110.1	H67A—C67—H67B	109.5
C25A—C26A—C27A	108.0 (10)	H67A—C67—H67C	109.5
H26C—C26A—H26D	108.4	H67B—C67—H67C	109.5
C27A—C26A—H26C	110.1	C68—C67—H67A	109.5
C27A—C26A—H26D	110.1	C68—C67—H67B	109.5
C26A—C27A—H27C	109.9	C68—C67—H67C	109.5
C26A—C27A—H27D	109.9	O7—C68—C67	114.5 (8)
H27C—C27A—H27D	108.3	O7—C68—H68A	108.6
C28A—C27A—C26A	108.9 (11)	O7—C68—H68B	108.6
C28A—C27A—H27C	109.9	C67—C68—H68A	108.6
C28A—C27A—H27D	109.9	C67—C68—H68B	108.6
C27A—C28A—H28C	109.7	H68A—C68—H68B	107.6
C27A—C28A—H28D	109.7	O7—C69—H69A	111.6
C27A—C28A—C29A	109.8 (11)	O7—C69—H69B	111.6
H28C—C28A—H28D	108.2	O7—C69—C70	100.9 (9)
C29A—C28A—H28C	109.7	H69A—C69—H69B	109.4
C29A—C28A—H28D	109.7	C70—C69—H69A	111.6
C28A—C29A—H29C	109.1	C70—C69—H69B	111.6
C28A—C29A—H29D	109.1	C69—C70—H70A	109.5
H29C—C29A—H29D	107.9	C69—C70—H70B	109.5
C30A—C29A—C28A	112.4 (12)	C69—C70—H70C	109.5
C30A—C29A—H29C	109.1	H70A—C70—H70B	109.5
C30A—C29A—H29D	109.1	H70A—C70—H70C	109.5
C25A—C30A—C29A	109.2 (11)	H70B—C70—H70C	109.5
C25A—C30A—H30C	109.8		
			
Co1—P1—N1—P2	−7.34 (8)	C27A—C28A—C29A—C30A	−56.8 (18)
Co1—P1—N1—C25	−175.6 (3)	C28A—C29A—C30A—C25A	55.7 (18)
Co1—P1—N1—C25A	156.7 (6)	C30A—C25A—C26A—C27A	61.9 (15)
Co1—P1—C1—C2	−59.3 (2)	P4—C43—C44—C45	176.7 (6)
Co1—P1—C1—C6	116.26 (18)	P4—C43—C48—C47	−176.0 (5)
Co1—P1—C7—C8	104.65 (19)	P4—C61—C62—C63	179.6 (8)
Co1—P1—C7—C12	−68.12 (19)	P4—C61—C66—C42	−178 (3)
Co1—P2—N1—P1	7.32 (8)	O2—P4—O4—Co2	41.2 (4)
Co1—P2—N1—C25	175.0 (3)	O2—P4—C43—C44	127.3 (5)
Co1—P2—N1—C25A	−156.9 (6)	O2—P4—C43—C48	−54.0 (7)
Co1—P2—C13—C14	65.15 (19)	O2—P4—C61—C62	118.3 (5)
Co1—P2—C13—C18	−111.36 (17)	O2—P4—C61—C66	−60.6 (19)
Co1—P2—C19—C20	57.5 (2)	O4—P4—O2—Co3	12.1 (4)
Co1—P2—C19—C24	−118.31 (19)	O4—P4—C43—C44	0.2 (5)
P1—P2—N1—C25	167.7 (3)	O4—P4—C43—C48	178.9 (6)
P1—P2—N1—C25A	−164.2 (6)	O4—P4—C61—C62	−115.0 (5)
P1—P2—C13—C14	−3.5 (2)	O4—P4—C61—C66	66.1 (19)
P1—P2—C13—C18	−179.98 (14)	C43—P4—O2—Co3	−109.1 (4)
P1—P2—C19—C20	119.73 (17)	C43—P4—O4—Co2	164.3 (3)
P1—P2—C19—C24	−56.1 (2)	C43—P4—C61—C62	−0.6 (6)
P1—N1—C25—C26	−153.6 (3)	C43—P4—C61—C66	−179.5 (19)
P1—N1—C25—C30	−28.3 (5)	C43—C44—C45—C46	−0.2 (11)
P1—N1—C25A—C26A	44.1 (12)	C44—C43—C48—C47	2.7 (12)
P1—N1—C25A—C30A	170.1 (7)	C44—C45—C46—C47	2.0 (12)
P1—C1—C2—C3	175.25 (18)	C45—C46—C47—C48	−1.3 (12)
P1—C1—C6—C5	−174.3 (2)	C46—C47—C48—C43	−1.0 (13)
P1—C7—C8—C9	−174.76 (18)	C48—C43—C44—C45	−2.0 (10)
P1—C7—C12—C11	175.24 (19)	C61—P4—O2—Co3	133.3 (4)
P2—P1—N1—C25	−168.3 (3)	C61—P4—O4—Co2	−80.2 (4)
P2—P1—N1—C25A	164.1 (6)	C61—P4—C43—C44	−115.0 (5)
P2—P1—C1—C2	10.2 (2)	C61—P4—C43—C48	63.7 (7)
P2—P1—C1—C6	−174.18 (15)	C61—C62—C63—C64	−4.0 (19)
P2—P1—C7—C8	42.9 (2)	C62—C61—C66—C42	3 (5)
P2—P1—C7—C12	−129.91 (16)	C62—C63—C64—C42	9 (3)
P2—N1—C25—C26	41.7 (5)	C64—C42—C66—C61	1 (5)
P2—N1—C25—C30	167.0 (3)	C66—C42—C64—C63	−7 (4)
P2—N1—C25A—C26A	−156.0 (6)	C66—C61—C62—C63	−1.4 (19)
P2—N1—C25A—C30A	−30.0 (13)	P3—C31—C32—C33	−175.7 (3)
P2—C13—C14—C15	−177.13 (17)	P3—C31—C36—C35	175.7 (3)
P2—C13—C18—C17	175.80 (19)	P3—C37—C38—C39	177.5 (17)
P2—C19—C20—C21	−176.0 (2)	P3—C37—C65—C41	−174 (2)
P2—C19—C24—C23	175.2 (2)	O1—P3—O6—Co3	10.3 (4)
N1—P1—C1—C2	48.6 (2)	O1—P3—C31—C32	−24.3 (3)
N1—P1—C1—C6	−135.85 (19)	O1—P3—C31—C36	160.0 (2)
N1—P1—C7—C8	−0.1 (2)	O1—P3—C37—C38	−15 (3)
N1—P1—C7—C12	−172.83 (17)	O1—P3—C37—C65	161 (3)
N1—P2—C13—C14	−42.3 (2)	O6—P3—O1—Co2	42.2 (4)
N1—P2—C13—C18	141.15 (18)	O6—P3—C31—C32	−153.2 (3)
N1—P2—C19—C20	162.71 (18)	O6—P3—C31—C36	31.1 (3)
N1—P2—C19—C24	−13.1 (2)	O6—P3—C37—C38	112 (3)
N1—C25—C26—C27	−178.2 (4)	O6—P3—C37—C65	−73 (3)
N1—C25—C30—C29	176.0 (3)	C31—P3—O1—Co2	−80.9 (3)
N1—C25A—C26A—C27A	−169.7 (8)	C31—P3—O6—Co3	134.1 (3)
N1—C25A—C30A—C29A	175.7 (9)	C31—P3—C37—C38	−131 (3)
C1—P1—N1—P2	−133.55 (10)	C31—P3—C37—C65	44 (3)
C1—P1—N1—C25	58.2 (3)	C31—C32—C33—C34	0.0
C1—P1—N1—C25A	30.5 (6)	C32—C31—C36—C35	0.0
C1—P1—C7—C8	−117.51 (19)	C32—C33—C34—C35	0.0
C1—P1—C7—C12	69.7 (2)	C33—C34—C35—C36	0.0
C1—C2—C3—C4	−0.4 (4)	C34—C35—C36—C31	0.0
C2—C1—C6—C5	1.3 (4)	C36—C31—C32—C33	0.0
C2—C3—C4—C5	0.6 (4)	C37—P3—O1—Co2	162.2 (13)
C3—C4—C5—C6	0.3 (4)	C37—P3—O6—Co3	−108.2 (12)
C4—C5—C6—C1	−1.2 (4)	C37—P3—C31—C32	89.8 (14)
C6—C1—C2—C3	−0.5 (3)	C37—P3—C31—C36	−86.0 (13)
C7—P1—N1—P2	110.63 (10)	C37—C38—C39—C40	0 (2)
C7—P1—N1—C25	−57.7 (3)	C38—C37—C65—C41	3 (4)
C7—P1—N1—C25A	−85.3 (6)	C38—C39—C40—C41	−6 (2)
C7—P1—C1—C2	166.97 (18)	C39—C40—C41—C65	9 (3)
C7—P1—C1—C6	−17.4 (2)	C40—C41—C65—C37	−8 (4)
C7—C8—C9—C10	0.5 (4)	C65—C37—C38—C39	1 (3)
C8—C7—C12—C11	2.2 (3)	P5—C49—C50—C51	−178.4 (3)
C8—C9—C10—C11	1.1 (4)	P5—C49—C54—C53	178.4 (3)
C9—C10—C11—C12	−1.0 (4)	P5—C55—C56—C57	−174.9 (6)
C10—C11—C12—C7	−0.6 (4)	P5—C55—C60—C59	174.4 (5)
C12—C7—C8—C9	−2.1 (3)	O3—P5—O5—Co3	19.5 (4)
C13—P2—N1—P1	132.65 (9)	O3—P5—C49—C50	−149.0 (3)
C13—P2—N1—C25	−59.6 (3)	O3—P5—C49—C54	32.6 (3)
C13—P2—N1—C25A	−31.5 (6)	O3—P5—C55—C56	−139.4 (4)
C13—P2—C19—C20	−79.2 (2)	O3—P5—C55—C60	43.8 (5)
C13—P2—C19—C24	105.0 (2)	O5—P5—O3—Co2	28.7 (4)
C13—C14—C15—C16	1.5 (3)	O5—P5—C49—C50	83.2 (3)
C14—C13—C18—C17	−0.8 (3)	O5—P5—C49—C54	−95.2 (3)
C14—C15—C16—C17	−1.1 (4)	O5—P5—C55—C56	−11.2 (5)
C15—C16—C17—C18	−0.2 (4)	O5—P5—C55—C60	171.9 (4)
C16—C17—C18—C13	1.2 (4)	C49—P5—O3—Co2	−94.0 (3)
C18—C13—C14—C15	−0.6 (3)	C49—P5—O5—Co3	142.2 (3)
C19—P2—N1—P1	−111.97 (11)	C49—P5—C55—C56	104.6 (4)
C19—P2—N1—C25	55.7 (3)	C49—P5—C55—C60	−72.2 (5)
C19—P2—N1—C25A	83.8 (6)	C49—C50—C51—C52	0.0
C19—P2—C13—C14	−161.32 (18)	C50—C49—C54—C53	0.0
C19—P2—C13—C18	22.2 (2)	C50—C51—C52—C53	0.0
C19—C20—C21—C22	1.1 (4)	C51—C52—C53—C54	0.0
C20—C19—C24—C23	−0.5 (4)	C52—C53—C54—C49	0.0
C20—C21—C22—C23	−1.6 (4)	C54—C49—C50—C51	0.0
C21—C22—C23—C24	1.1 (4)	C55—P5—O3—Co2	151.9 (3)
C22—C23—C24—C19	0.0 (4)	C55—P5—O5—Co3	−103.8 (3)
C24—C19—C20—C21	0.0 (4)	C55—P5—C49—C50	−32.7 (3)
C25—C26—C27—C28	−54.8 (7)	C55—P5—C49—C54	148.8 (3)
C26—C25—C30—C29	−58.3 (7)	C55—C56—C57—C58	−0.4 (12)
C26—C27—C28—C29	56.3 (7)	C56—C55—C60—C59	−2.4 (9)
C27—C28—C29—C30	−58.8 (7)	C56—C57—C58—C59	−0.7 (13)
C28—C29—C30—C25	59.6 (7)	C57—C58—C59—C60	0.2 (11)
C30—C25—C26—C27	55.7 (7)	C58—C59—C60—C55	1.4 (10)
C25A—C26A—C27A—C28A	−61.6 (16)	C60—C55—C56—C57	1.9 (9)
C26A—C25A—C30A—C29A	−58.8 (16)	C68—O7—C69—C70	−151.7 (11)
C26A—C27A—C28A—C29A	58.6 (16)	C69—O7—C68—C67	−166.6 (12)

Symmetry code: (i) −*x*+1, −*y*, −*z*.

### Bis[N,N-bis(diphenylphosphanyl)cyclohexanamine-κ2P,P']dichloridocobalt(III) tris(µ-diphenylphosphinato-κ2O:O')bis[chloridocobaltate(II)]–dichloromethane–diethyl ether (1/1.189/0.811) Hydrogen-bond geometry (Å, º)

*Cg*1, *Cg*2 and *Cg*3 are the centroids of the C37–C41/C65, C42/C61–C64/C66 and C19–C24 rings, respectively.

**Table:**

*D*—H···*A*	*D*—H	H···*A*	*D*···*A*	*D*—H···*A*
C2—H2···Cl1^i^	0.95	2.66	3.174 (2)	114
C6—H6···Cl4^i^	0.95	2.95	3.668 (4)	133
C17—H17···O7	0.95	2.48	3.314 (8)	146
C24—H24···Cl2^ii^	0.95	2.96	3.682 (5)	133
C27—H27*B*···*Cg*1	0.99	2.97	3.834 (11)	147
C53—H53···*Cg*2^iii^	0.95	2.98	3.787 (8)	143
C60—H60···*Cg*3^ii^	0.95	2.72	3.626 (8)	159
C29*A*—H29*C*···*Cg*2^ii^	0.99	2.69	3.57 (2)	148

Symmetry codes: (i) −*x*+1, −*y*, −*z*; (ii) −*x*+1, −*y*, −*z*+1; (iii) *x*−1, *y*, *z*.
